# Malignant Hypertension in Association with Low Estrogen Dose Oral Contraceptives: Case Report and Review of Literature

**DOI:** 10.7759/cureus.2978

**Published:** 2018-07-13

**Authors:** Danial Mir, Arash Ardabilygazir, Sonia Afshariyamchlou, Issac Sachmechi

**Affiliations:** 1 Medicine, Icahn School of Medicine at Mount Sinai/Queens Hospital Center, New York, USA; 2 Internal Medicine/Endocrinology, Icahn School of Medicine at Mount Sinai/Queens Hospital Center, Jamaica, USA; 3 Internal Medicine, Icahn School of Medicine at Mount Sinai/Queens Hospital Center, New York, USA

**Keywords:** malignant hypertension, high blood pressure and oral contraceptive pills, high-dose estrogen and blood pressure, oral contraceptive pills (ocp)

## Abstract

Malignant hypertension (MH) has been described in association with high-dose (50 - 100 mcg) estrogen oral contraceptive pills (OCPs). Although the rise in blood pressure (BP) is usually mild, some women will have a more significant increase in BP, and hypertensive emergencies may very rarely occur. We present a 21-year-old Caucasian female with a past medical history of fibromyalgia and family history of hypertension (both grandparents) who was admitted with a three-day history of headache and blurring of vision in her left eye with a BP of 210/150. Her medications, which were continued on admission, included tramadol, 100 mg twice daily (bid), and low-dose estrogen OCP. During the hospital course, she received different antihypertensive medications and her hypertension was controlled. A diagnosis of MH due to OCP was made. All antihypertensive medications were stopped, except metoprolol, and the patient was discharged home on metoprolol with a BP of 107/55 mmHg. On follow-up in the medical clinic three months later, her visual disturbances had completely resolved and her BP was 98/56 mmHg.

One-third of patients aged 15 - 44 years old who develop MH are likely to be on high-dose estrogen OCP. As far as we know, our case is the third documented case of MH occurring in patients on low-dose estrogen OCP. Chronic use of oral contraceptives will slightly increase the systemic BP in most women. It is advisable to avoid OCP in high-risk patients and do regular BP checks on patients on OCP. In patients presenting with hypertension or MH while on OCP, the OCP should be discontinued.

## Introduction

For millions of women, oral contraceptive pills (OCPs) are an effective way to prevent an unwanted pregnancy, get irregular periods back on track, and help clear up problems due to hormonal shifts, such as acne. For the most part, birth control pills are safe and have merely minor potential side effects. However, OCPs may be associated with an increased risk of myocardial infarction (MI). Because MI is an extremely rare event in otherwise healthy women of reproductive age, even a doubling of the risk would result in an extremely low attributable risk [1]. An increase in the risk of venous thromboembolic (VTE) disease is seen with both high and low-dose estrogen OCP preparations. Although the reduction in steroid content of OCPs has improved the safety and side effect profile of the pill, the increased risk of venous thrombosis has not been eliminated. The risk is affected by patient age, weight, and thrombophilia status. The type of progestin also affects the risk [2-3].

Malignant hypertension (MH) has been described in association with high-dose (50 - 100 mcg) estrogen OCPs since the publication of Weir et al. in 1971 [4]. Although the rise in blood pressure (BP) is usually mild, some women will have a more significant increase in BP, and hypertensive emergencies may very rarely occur. Development of hypertension is more likely to occur in patients who developed hypertension during a prior pregnancy or who have a family history of hypertension [5]. Although 5% of women on OCP develop hypertension, the incidence of MH in this group of patients is unknown. Malignant phase hypertension may occur within weeks of commencing therapy or after several years. Despite the fact that recent preparations contain lower doses of estrogen, there is no room for complacency when giving OCP. Close medical supervision and regular blood pressure measurements during OCP treatment are mandatory. We present a case of MH developing in a patient within two years of commencing OCP containing low-dose (30 mcg) estrogen.

## Case presentation

**Figure 1:**
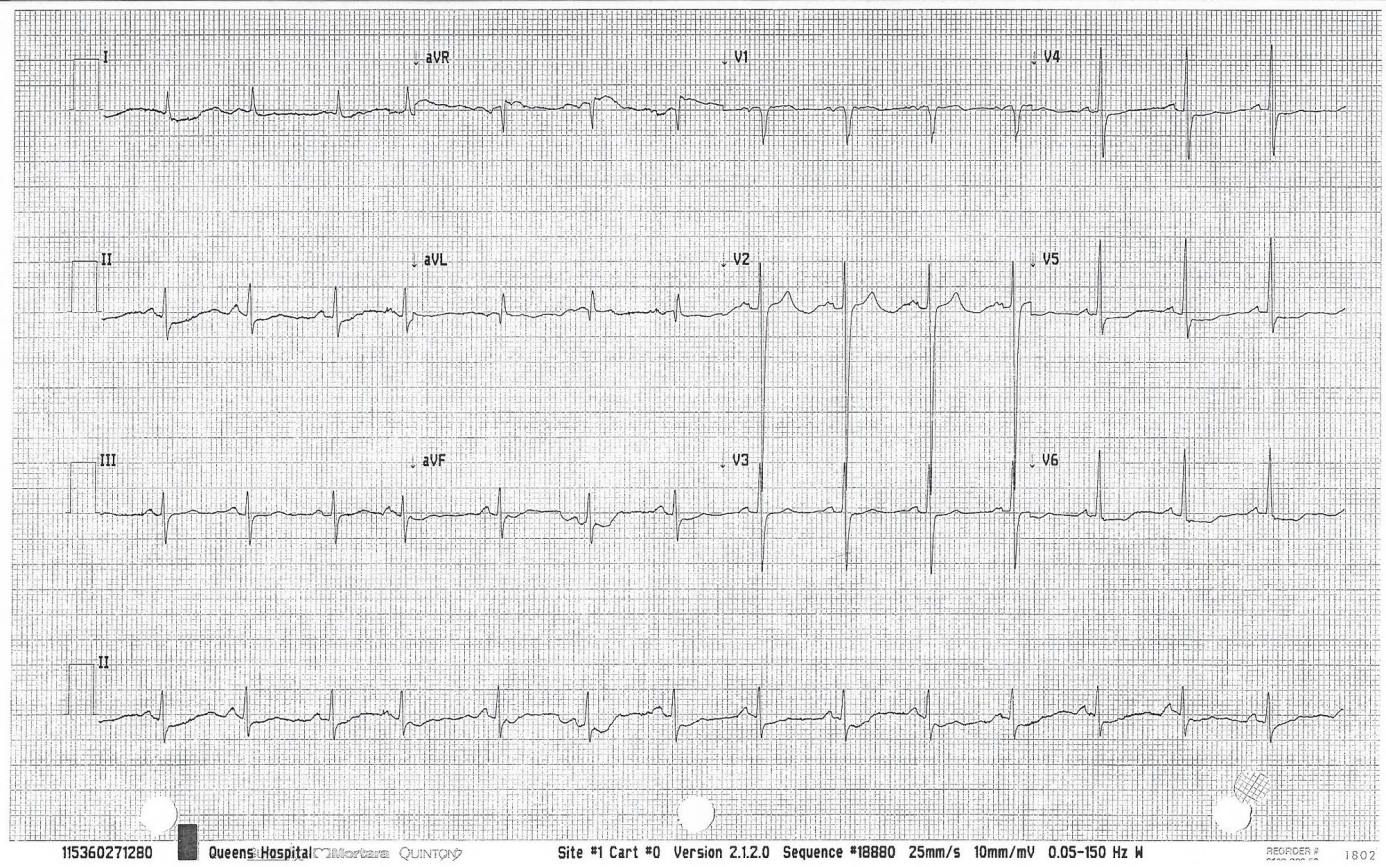
Left ventricular hypertrophy

A 21-year-old Caucasian female with a past medical history of fibromyalgia and a family history of hypertension (both grandparents) was admitted with a three-day history of headache and blurring of vision in her left eye. Her BP was 210/150 and physical examination was essentially normal, apart from the visual acuity of 6/4 (right eye) and 6/1 (left eye) with papilledema. Laboratory workup revealed a normal blood count, renal function, liver function, serum angiotensin-converting enzyme (ACE), 24-hour urinary catecholamine/cortisol, chest x-ray, abdominal/renal ultrasound, sestamibi scan, computed tomography (CT) of the head/renal angiogram, and magnetic resonance imaging (MRI) of the aorta/chest/ovaries. The renin-aldosterone level was raised with a recumbent aldosterone/renin of 8.5 ng/dl and 908 ng/ml/hr, respectively, as well as a standing aldosterone/renin of 19.30 ng/dl and 1964 ng/ml/hour (hr), respectively. An electrocardiogram was significant for left ventricular hypertrophy (LVH) (Figure 1), an echo showed LVH and an ejection fraction of 45%, and a transesophageal echocardiogram confirmed an incidental leiomyomatous interventricular septum. Her medications, which were continued on admission, included tramadol, 100 mg twice daily (bid), and low-dose estrogen OCP. During the hospital course, Labetalol, 200 mg bid, was started. The BP remained at 165/105 mm Hg with a heart rate (HR) of 58 beats per min (bpm). Labetalol was discontinued and Moxonidine, 200 mcg, Indapamide, 5 mg, and amlodipine, 10 mg (all once daily), were initiated. By hospital day 15, the BP remained elevated at 170/100 mmHg with HR at 88 bpm. The OCP and amlodipine were stopped and metoprolol, 25 mg bid, was started. By hospital day 32, her BP was 108/60 mmHg. A diagnosis of MH due to OCP was made, and all anti-hypertensive medications were stopped except for the metoprolol. The patient was discharged home on metoprolol with a BP of 107/55 mmHg. On follow-up in the medical clinic three months later, the visual disturbances had completely resolved and her BP was 98/56 mmHg. Repeat renin/aldosterone was normal. Her visual acuity was 6/4 (right) and 6/5 (left). The metoprolol was stopped, and a BP check one year later was 103/58 mmHg.

## Discussion

One-third of patients aged 15-44 years old who develop MH are likely to be on high-dose estrogen OCP. As far as we know, our case is the third documented case of MH occurring in a patient on low-dose estrogen OCP. The risk factors for developing MH include the use of high-dose estrogen OCP, hypertension in pregnancy and family members, diabetes mellitus, obesity, smoking, alcohol abuse, and age greater than 35 years. Mechanisms of development of MH include an increased hepatic production of renin substrate, arteriolar fibrinoid necrosis, thrombosis, and renal infarction. The singular most important predictor of resolution of MH is the presence of renal impairment. Although the presence of LVH suggests long-term hypertension in our patient, the absence of renal impairment suggests an excellent long-term prognosis. MH can occur within eight weeks to eight years of commencing OCP. The 10-year survival is 90% if there are no other comorbidities compared with 50% in patients with renal impairment. In this patient, the MH caused by OCP induced an increase in renin secretion, causing the secondary elevation of aldosterone. Her hypertension was fully resolved within three months of stopping the OCP.  

In the review of the literature (Table 1), Harris et al. described a case of MH associated with oral contraceptive therapy. The patient had never been pregnant and had no family history of hypertension. After one year of antihypertensive therapy, the patient developed symptoms of postural hypotension. Therapy was discontinued and the patient’s blood pressure subsequently returned to normal [6]. Zacherle et al. described a case with severe hypertension that developed while the patient was on OCP. Antihypertensive agents controlled the blood pressure satisfactorily and the BP remained normal after withdrawal of all therapy. Reintroduction of a sequential oral contraceptive agent three years later again resulted in severe hypertension associated on this occasion with early hypertensive retinopathy and irreversible renal failure [7]. Dunn et al. presented a case of a young lady with a history of hypertension and being overweight who developed MH after taking oral contraceptive preparations for two years. Control of her BP was obtained by the withdrawal of the oral contraceptive agent and antihypertensive therapy [8]. Weir et al. found the increase in systolic blood pressure to be the same in women taking either low-dose or high-dose estrogen oral contraceptive preparations [4]. The mechanisms responsible for the hypertensive effect of oral contraceptives are incompletely understood. Both estrogen and progesterone appear to contribute in a dose-dependent fashion. The renin-angiotensin system (RAS) may be involved since estrogen stimulates the hepatic production of renin substrate (angiotensinogen) [9]. Early epidemiologic studies using high-dose estrogen found a mean elevation in BP of 3 to 5 mmHg, with approximately 5% of women developing overt hypertension [10]. A subsequent prospective cohort study of over 68,000 women on lower doses of estrogen demonstrated that, as compared with women who had never used oral contraceptives, the age-adjusted relative risk of developing hypertension was 1.5 for current use and 1.1 for past use [11].

**Table 1:** Summary of Cases Reviewed in the Literature HTN: hypertension; OCPs: oral contraceptive pills; BP: blood pressure.

First Author (Ref.)	Year	Case	Description
Harris [6]	1969	27-year-old female	-No previous history of HTN or renal disease/No family history of HTN -Taking OCPs for 30 months which led to malignant HTN (BP = 220/150) -Within a month, her BP had fallen to 200/110 mm Hg after taking antihypertensive medications and stopped taking OCPs
Zacherle et al. [7]	1972	29-year-old female	-No previous history of HTN or renal disease/No family history of HTN -First time taking OCP was in 1967 and after one year, BP increased to 220/150, and by stopping OCP and taking antihypertensive medication, high BP dropped to normal after three months. Second time taking OCP was in 1970, and after one year, malignant HTN (BP = 250/190) led to irreversible renal failure
Dunn et al. [8]	1975	26-year-old female	-History of gestational HTN in 1960. Started OCP in 1964 and got malignant HTN (BP = 230/150) in 1966 -High BP was rapidly controlled after discontinuing OCP and taking antihypertensive medications
Weir et al. [4]	1971-1974	A prospective study of 66 women	-High systolic BP after one year (between 115.1 +/- 1.3), high diastolic BP at the end of two years -Discontinuance of OCPs resulted in the return of BP to pretreatment levels within three months -No cases of severe or malignant HTN among patients.

## Conclusions

The chronic use of oral contraceptives will slightly increase the systemic BP in most women. It is advisable to avoid OCP in high-risk patients and do regular BP checks on patients on OCP. In patients presenting with hypertension or MH while on OCP, the OCP should be discontinued with a follow-up of the BP before an extensive workup is done. 
